# Association between vitamin D and ovarian cancer development in *BRCA1* mutation carriers

**DOI:** 10.18632/oncotarget.27803

**Published:** 2020-11-10

**Authors:** Tanja Pejovic, Sonali Joshi, Shawn Campbell, Sarah Thisted, Fuhua Xu, Jing Xu

**Affiliations:** ^1^Division of Gynecologic Oncology, Department of Obstetrics and Gynecology, School of Medicine, Oregon Health & Science University, Portland, Oregon, USA; ^2^Division of Reproductive Endocrinology, Department of Obstetrics and Gynecology, School of Medicine, Oregon Health & Science University, Portland, Oregon, USA; ^3^Present address: College of Health and Human Services, Northern Arizona University, Flagstaff, Arizona, USA; ^4^Division of Reproductive & Developmental Sciences, Oregon National Primate Research Center, Oregon Health & Science University, Beaverton, Oregon, USA

**Keywords:** epithelial ovarian cancer, *BRCA1* mutation carriers, vitamin D, cancer prevention

## Abstract

Objective: Women with inherited mutations in *BRCA1* gene have a high (40–70%) genetic risk of developing ovarian cancer. Epidemiological studies suggest an inverse correlation between serum vitamin D (VD) levels and the risk of ovarian cancer, but there is a lack of data from *BRCA1* mutation (*BRCA1*^mut^) carriers. Therefore, we investigated VD levels and actions in cancer free women with *BRCA1* mutations.

Materials and Methods: Blood, ovary and fallopian tube samples were collected from healthy pre-menopausal women with *BRCA1*^mut^ and without BRCA1 mutations (*BRCA*^wt^). Serum calcifediol (major circulating form of VD) concentrations were measured by electrochemiluminescence immunoassay. Immunohistochemistry was performed on paraffin-embedded ovarian and fallopian tube sections to determine vitamin D receptor (VDR) expression. Ovarian surface epithelial cells (OSEs) from *BRCA1*^mut^ carriers were cultured with or without calcitriol supplementation for 72 hrs. VDR protein levels, cell proliferation and cell viability were analyzed.

Results: *BRCA1*^mut^ women had lower serum calcifediol levels compared to *BRCA*^wt^ women (*p* = 0.003). VDR protein expression was evident in ovarian and the fallopian tube epithelium of *BRCA*^wt^ patients, but was reduced in *BRCA1*^mut^ women. Calcitriol (biologically active VD) supplementation elevated VDR expression in cultured *BRCA1*^mut^ OSEs (*p* = 0.005) and decreased cell proliferation rates in a dose-dependent manner without inducing apoptosis.

Conclusions: VD biosynthesis and signaling via VDR in the ovarian and fallopian tube epithelium are impaired in *BRCA1*^mut^ women. VD treatment may limit *BRCA1*^mut^ epithelial cell proliferation without affecting cell viability, providing a rationale for exploring the potential for VD in ovarian cancer prevention in *BRCA1*^mut^ carriers.

## INTRODUCTION

Epithelial ovarian cancer (EOC) is a lethal disease with 5-year survival rates of 40% [1]. Only 15% of EOC patients are diagnosed with Stage I disease and have favorable 5 year survival rate of 92% [2]. However, lack of early detection methods leads to late stage disease at diagnosis and poor outcomes in majority of the patients. Women at a high risk for EOC are offered regular pelvic exams in combination with transvaginal ultrasound and a blood test for CA-125 tumor marker, but these strategies have failed to significantly improve patient survival [3]. Developing strategies to identify women with a high risk of developing EOC and to prevent tumor development is critical for reducing EOC-related mortality.

About 24% of EOCs are associated with hereditary conditions [4] and inherited heterozygous mutations in breast cancer susceptibility gene (*BRCA*) 1 and 2 account for 65–85% of hereditary ovarian tumors [5]. *BRCA1* maintains genomic stability and exhibits tumor suppressive activities by regulating DNA repair, chromatin reorganization, transcription and ubiquitination [6]. *BRCA1* mutation (*BRCA1*^mut^)-associated EOC is typically characterized by serous histology [7], and is more likely to be high grade and advanced stage [8]. Women with inherited *BRCA1*^mut^ have a 44% probability of developing EOC by age 80 compared to a 2% probability in the general population [9], and also have an increased probability of early onset of EOC [10]. To date, the only reliable risk-reducing method is surgery, i. e., bilateral salpingo-oophorectomy [11], which involves high financial and emotional costs. Based on a cumulative EOC risk of 0.55% to age 35 for *BRCA1*^mut^ carriers, an international registry study recommended bilateral salpingo-oophorectomy before age 40 to maximize prevention and to minimize adverse effects [12]. For pre-menopausal women, removing ovaries is associated with early menopause and infertility, and an increase in cardiovascular and neurological disease and osteoporosis [13]. Thus, there is an urgent need to develop other non-invasive and cost-effective methods to prevent EOC, particularly in young women carrying *BRCA1*^mut^.

Vitamin D (VD), a steroid hormone initially identified for its role in maintaining calcium homeostasis, exhibits pleotropic functions [14]. VD (cholecalciferol) is primarily produced in the skin upon UV light exposure, which is converted to calcifediol (major circulating form of VD) in the liver and then to calcitriol (biologically active form of VD) in the kidney [14]. VD binds to VD receptor (VDR), a member of the nuclear receptor superfamily, to activate downstream signaling and regulates expression of genes critical for cell proliferation, differentiation and apoptosis [14]. VDR expression has been reported in the ovary [15] and the fallopian tubes [16], from which EOC originates [17].

VD regulates expression of genes involved in the major hallmarks of cancer [18]. Epidemiological studies have demonstrated an inverse correlation between circulating calcifediol concentrations and cancer incidence in ovarian and other cancers [19, 20]. A Mendelian randomization study showed that genetically abated VD production was associated with increased risk of ovarian cancer in European women carrying specific single nucleotide polymorphisms (SNPs) of genes critical for VD biosynthesis [21]. Absence of VDR expression in *BRCA1*^mut^ breast tumors was associated with impaired overall patient survival [22] and a shorter disease-free interval [23], suggesting a possible relationship between VD signaling and cancer development in *BRCA1*^mut^ carriers. However, VD levels and actions in EOC development have not been reported in women with *BRCA1*^mut^.

Considering the regulatory effects of VD on cell proliferation, we investigated the potential for VD in the prevention of EOC in *BRCA1*^mut^ carriers. We sought to determine circulating VD levels and VDR expression in ovarian surface epithelial (OSE)/fallopian tube epithelial (FTE) cells in healthy women with *BRCA1*^mut^. We also examined whether VD supplementation can regulate VDR expression and the proliferation of OSE cells from healthy *BRCA1*^mut^ carriers.

## RESULTS

### Reduced serum VD levels in healthy women with *BRCA1*^mut^

We analyzed serum calcifediol levels in healthy pre-menopausal women with *BRCA1*^mut^ and *BRCA*^wt^ patients. The *BRCA1*^mut^ types and related functional consequences are summarized in Table 1. Calcifediol, the major circulating form of VD with a half-life of 2–3 weeks, is a common measure of systemic VD levels [24]. Calcifediol was detectable in all serum samples. Although ages were comparable between *BRCA1*^mut^ carriers and *BRCA*^wt^ patients (36.1 ± 1.7 versus 37.1 ± 1.4 years; *p* = 0.33), *BRCA1*^mut^ carriers had ~33% lower (*p* = 0.003) serum calcifediol concentrations than those of *BRCA*^wt^ patients (29.4 ± 2.3 versus 44.1 ± 4.0 ng/ml). Serum calcifediol level of 20–29.9 ng/ml is defined as VD insufficiency [25]. While ~92% (12 of 13) of *BRCA*^wt^ patients had healthy levels of VD (> 30 ng/ml serum calcifediol), 54% (6 of 11) of *BRCA1*^mut^ carriers exhibited VD insufficiency (21.7–27.7 ng/ml serum calcifediol) (Table 2).

**Table 1:** Summary of BRCA1 mutations in cancer-free women

Experiment	Patient	*BRCA1* mutation	Mutation Type	Clinical Significance	Functional consequence
Serum assay and immunohisto-chemistry	1	c.3916_3917del	Frameshift	Pathogenic	Truncated Protein
	2^*^	c.2722 G>T	Nonsense	Pathogenic	Truncated Protein
	3	c.5266dupC	Frameshift	Pathogenic	Truncated Protein
	4	c.1556del	Frameshift	Pathogenic	Truncated protein
	5	c.4065_4068del	Frameshift	Pathogenic	Truncated Protein
	6^*^	c.4065_4068del	Frameshift	Pathogenic	Truncated Protein
	7	c.66_67AG [1]	Frameshift	Pathogenic	Truncated Protein
	8	c.2035A>T	Nonsense	Pathogenic	Truncated Protein
	9^*^	c.66_67AG [1]	Frameshift	Pathogenic	Truncated Protein
	10	c.213-11T>G	Aberrant Splicing	Pathogenic	Nonsense mediated mRNA decay
	11^*^	c.2722G>T	Nonsense	Pathogenic	Truncated Protein
Ovarian surface epithelial cell culture	1	c.3112G>T	Nonsense	Pathogenic	Truncated protein
	2	c.66dupA	Frameshift	Pathogenic	Truncated Protein
	3	c.851_852del	Frameshift	Pathogenic	Truncated protein

^*^Ovarian and fallopian tube tissue from these patients were used for immunohistochemistry experiment.

**Table 2:** Serum 25-hydroxyvitamin D3 (calcifediol) levels in pre-menopausal cancer-free women with BRCA1 (BRCA1^mut^) or without (control) germline mutations in BRCA1 (BRCA^wt^)

	Patient	Age (years)	Calcifediol (ng/ml)
*BRCA*^wt^	1	41	56.96
	2	44	45.94
	3	30	46.06
	4	37	53.35
	5	43	45.82
	6	35	69.80
	7	34	33.00
	8	28	34.25
	9	35	40.75
	10	42	34.65
	11	36	61.35
	12	43	14.69
	13	34	36.39
*BRCA1*^mut^	1	41	23.44
	2	45	30.32
	3	30	22.12
	4	36	33.47
	5	43	23.46
	6	35	21.71
	7	32	45.99
	8	28	38.50
	9	33	27.66
	10	41	30.09
	11	33	26.53

### Reduced VDR expression in OSE and FTE in healthy women with *BRCA1*^mut^

**Figure 1:**
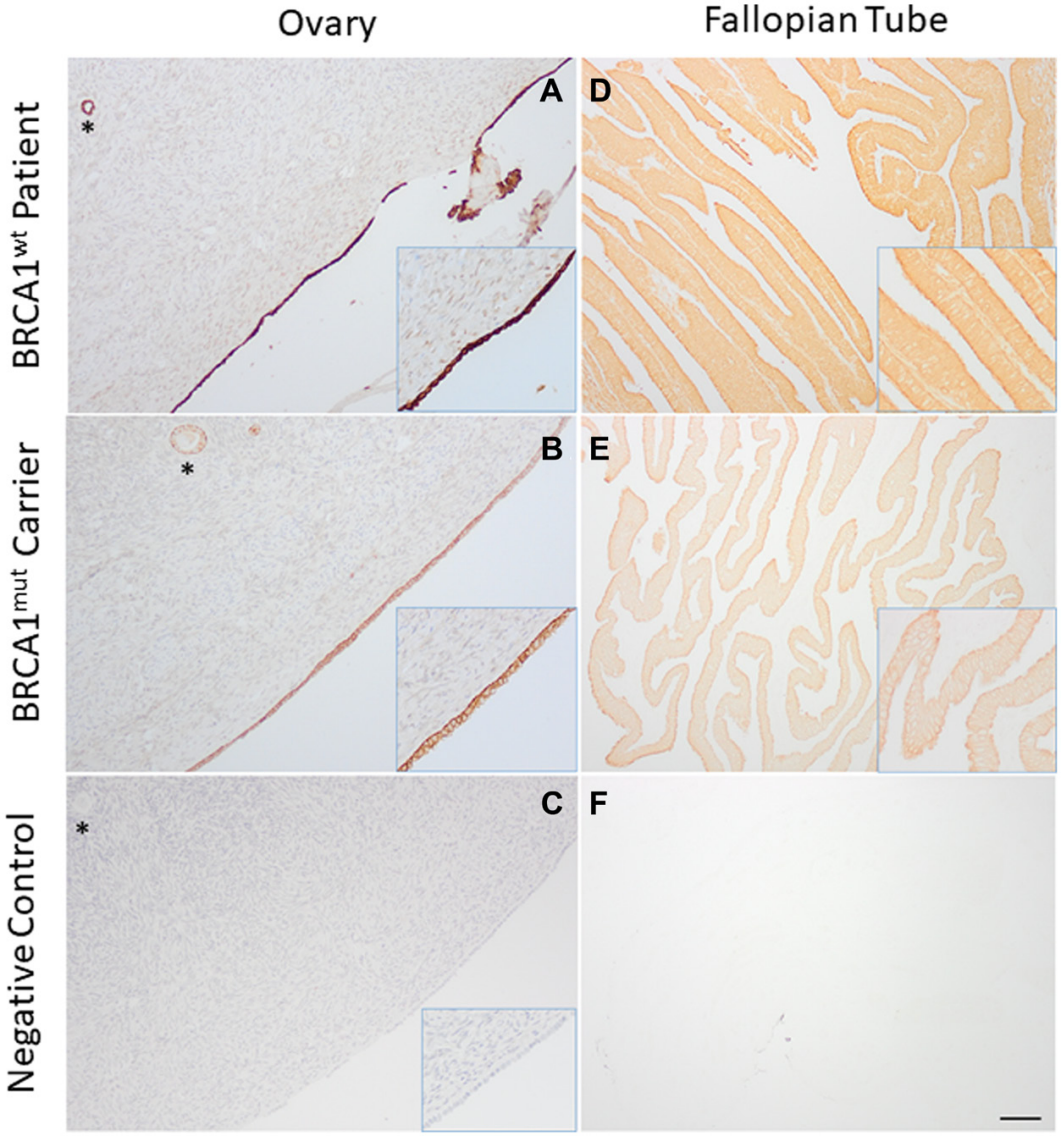
Vitamin D receptor (VDR) expression in the ovary and fallopian tube. Ovarian surface (**A** and **B**) and fallopian tube (**D** and **E**) epithelial cells in healthy women with *BRCA1* mutations (*BRCA1*^mut^; A and D) and women without *BRCA* mutations (*BRCA*^wt^; B and E) exhibited positive immunostaining of VDR (brown in representative sections). Immunohistochemistry was performed with (A and B) or without (D and E) counterstain hematoxylin to allow for visualization of VDR nuclear and cytoplasmic staining. Phosphate-buffered saline was used instead of anti-VDR antibody for the negative control ovarian (**C**) and fallopian tube (**F**) sections. Immunohistochemistry was performed with (C) or without (F) counterstain hematoxylin. ^*^ovarian follicle. Scale bar = 150 μm for A–F, and 75 μm for the insert (higher magnification).

VD is known to regulate cellular VDR expression [26] and is essential for the biological actions of VD [14]. Therefore, VDR protein levels in OSE and FTE were examined in healthy *BRCA1*^mut^ and *BRCA*^wt^ patients. VDR immunostaining (brown) was detected in the nucleus (DNA-bound) and cytoplasm (free form) of OSE (Figure 1A and 1B) and FTE (Figure 1D and 1E) cells, in addition to granulosa cells of ovarian follicles known for VDR expression (Figure 1A and 1B) [27]. VDR staining was intense in OSE and FTE cells, as well as in granulosa cells of ovarian follicles, in *BRCA*^wt^ patients (Figure 1A and 1D). In contrast, VDR staining intensity was reduced in *BRCA1*^mut^ carriers (Figure 1B and 1E). VDR immunostaining was absent in the negative control ovarian and fallopian tube sections with and without counterstaining, respectively (Figure 1C and 1F).

### Increased VDR protein levels in *BRCA1*^mut^ OSE in response to VD treatment

**Figure 2:**
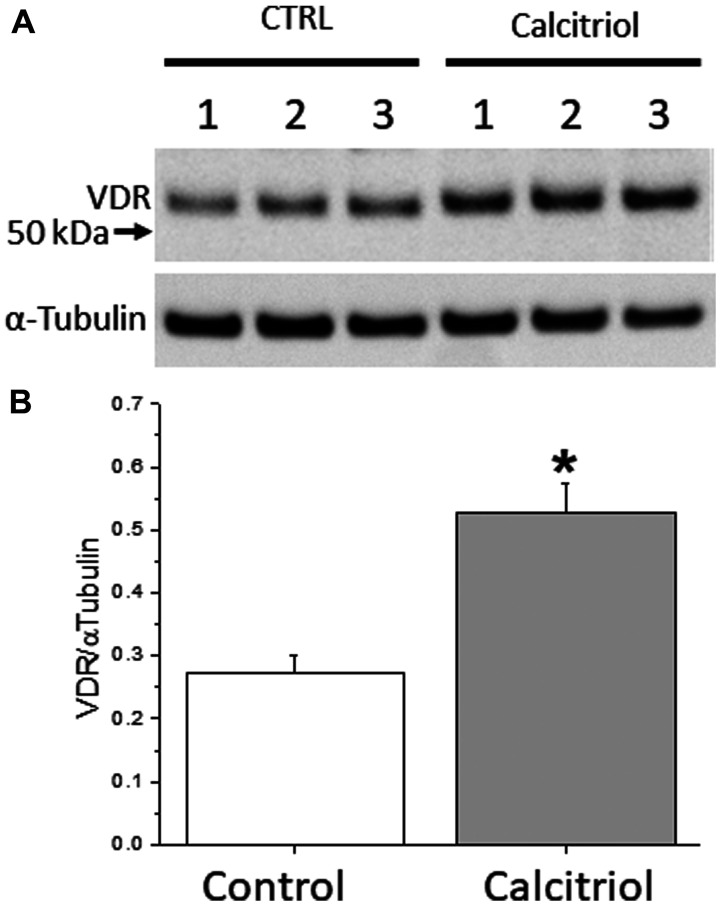
Vitamin D receptor (VDR) expression in cultured ovarian surface epithelial (OSE) cells. OSE cells derived from 3 healthy women with different germline *BRCA1* mutations (patient 1–3) were cultured without (control) or with 5 nM calcitriol supplementation in the medium for 72 hrs. VDR protein levels were analyzed by Western blot (**A**) followed by densitometry analysis (**B**). The α-Tubulin served as the loading control. ^*^significant difference between culture groups, *p* < 0.05. Data are presented as the mean ± SEM.

To further examine VD regulated VDR expression, OSE cells from 3 healthy women with different germline *BRCA1*^mut^ (Table 1) were cultured without (control) or with calcitriol, the biologically active form of VD that binds to VDR and activates downstream events [14]. VDR protein was detectable in OSE cells from all 3 *BRCA1*^mut^ patients after 72 hrs of culture without calcitriol supplementation (Figure 2A). VDR protein levels in *BRCA1*^mut^ OSE cells treated with calcitriol were elevated by ~95% relative to those of cells cultured under control conditions (VDR/αTubulin = 0.53 ± 0.05 versus 0.27 ± 0.03; *p* = 0.005) (Figure 2B).

### Reduced *BRCA1*^mut^ OSE cell proliferation in response to VD treatment

**Figure 3:**
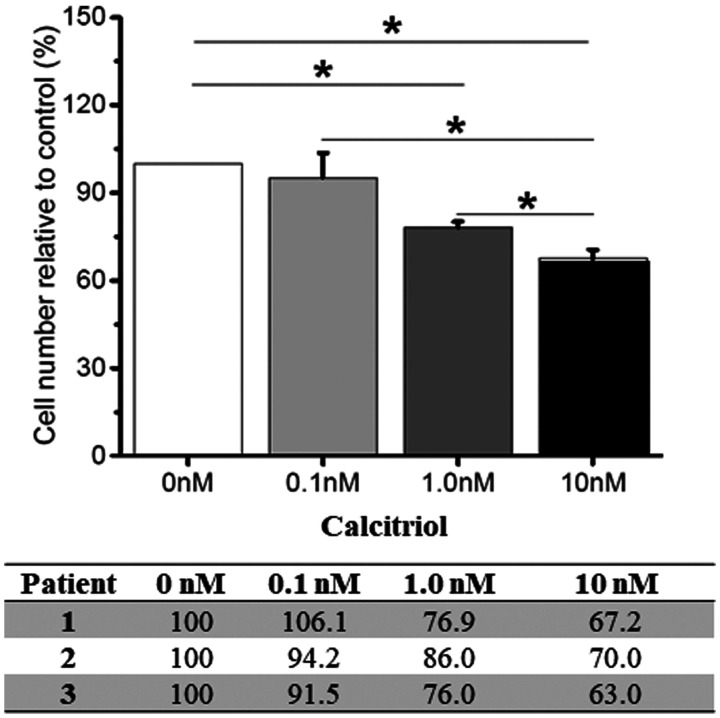
Vitamin D-regulated proliferation of cultured ovarian surface epithelial (OSE) cells. OSE cells derived from 3 healthy women with different germline *BRCA1* mutations (patient 1–3) were cultured with 0 (control), 0.1, 1.0 or 10 nM calcitriol supplementation in the medium for 72 hrs. Cells were counted and cell proliferation was presented as the ratio (%) of number of cells in the calcitriol culture to those of the control (0 nM) culture. Data from individual patients were presented in the table. ^*^significant difference between culture groups, *p* < 0.05. Data are presented as the mean ± SEM.

VD is known to attenuate cell proliferation in cultured ovarian cancer cell lines by inducing cell cycle arrest [28]. Therefore, a dose-response experiment was performed in OSE cells from 3 healthy women with different germline *BRCA1*^mut^ to examine cell proliferation in response to 72 hrs of calcitriol supplementation. The number of cultured *BRCA1*^mut^ OSE cells decreased (*p* < 0.05) with the calcitriol level as low as 1 nM, and further declined with the calcitriol dose increased to 10 nM (Figure 3). Similar dose-dependent cell proliferation patterns were observed in cultured *BRCA1*^mut^ OSE cells from all 3 patients regardless of their *BRCA1*^mut^ types (Figure 3).

### Unchanged *BRCA1*^mut^ OSE cell viability upon VD treatment

**Figure 4:**
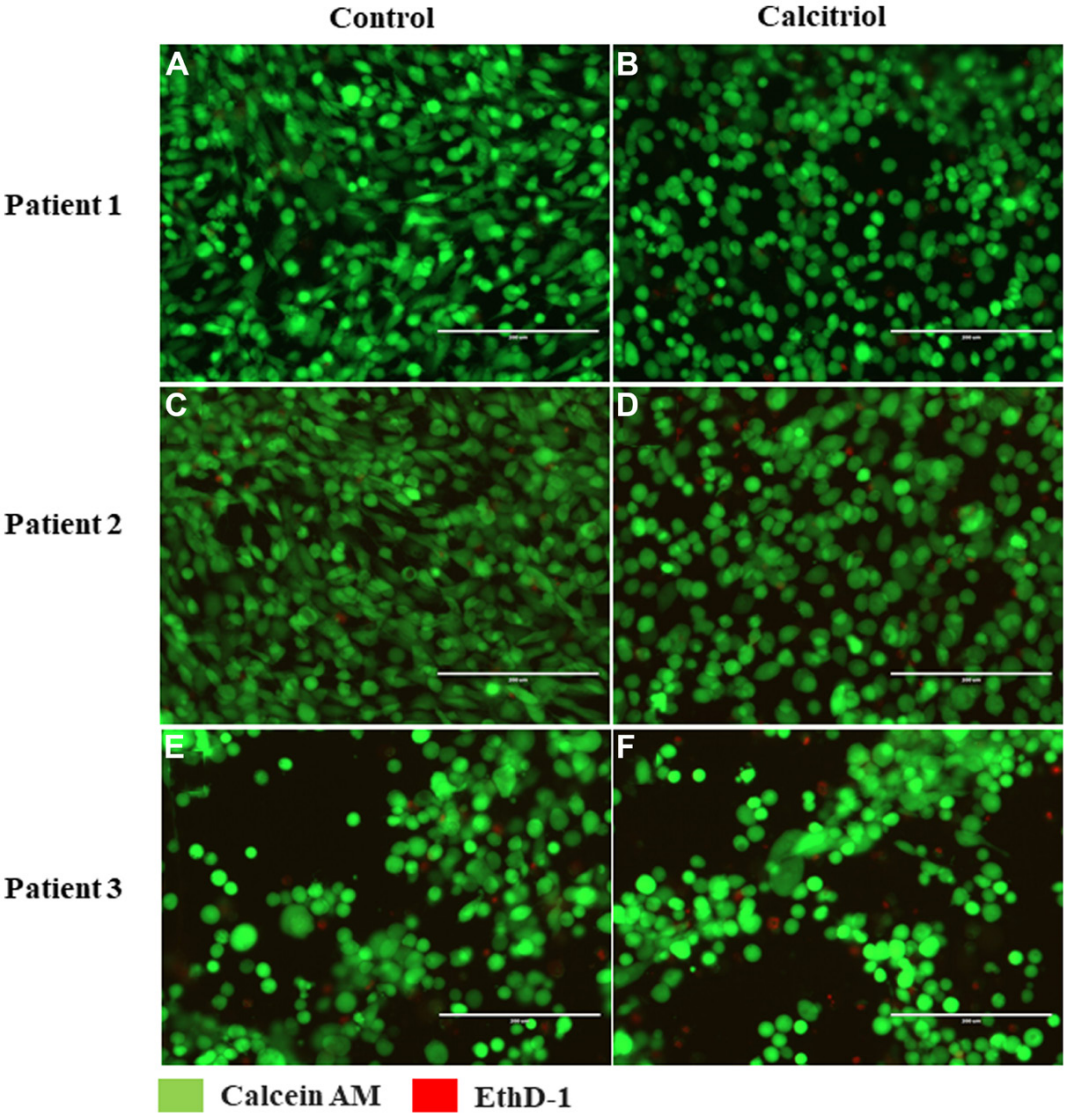
Vitamin D-regulated viability of cultured ovarian surface epithelial (OSE) cells. OSE cells derived from 3 healthy women with different germline *BRCA1* mutations (patient 1–3) were cultured without (control (**A**, **C**, **E**)) or with 5 nM calcitriol supplementation (**B**, **D**, **F**) in the medium for 72 hrs. Cell viability was assessed by live/dead cell analysis via incubation with calcein AM (green; live cells) and EthD-1 (red; dead cells). Scale bar = 200 μm.

As high-dose VD can induce apoptosis in cultured ovarian cancer cell lines [29], cytotoxic effects of VD were evaluated in OSE cells from 3 healthy women with different germline *BRCA1*^mut^ cultured without (control) or with calcitriol. After 72 hrs of culture, the majority of *BRCA1*^mut^ OSE cells were alive (green) regardless of *BRCA1*^mut^ types of patients or cell culture conditions (Figure 4). The numbers of dead (red) *BRCA1*^mut^ OSE cells were comparable between control and calcitriol treatment group (11.2 ± 0.8 versus 11.1 ± 0.5 cells; *p* = 0.45) (Figure 4).

## DISCUSSION

Women with *BRCA1*^mut^ face a difficult decision regarding risk-reducing surgery. Although bilateral salpingo-oophorectomy is associated with a substantial reduction in the risk of ovarian, fallopian tube, or peritoneal cancer in *BRCA1*^mut^ carriers [30], the incidence of primary peritoneal carcinoma has been reported after surgery [31]. As FTE has been identified as a site of origin for many serous EOC, so-called “staged” surgery with removal of the fallopian tubes at an earlier age, followed by removal of ovaries later before the age of 45, is being evaluated in clinical trials [32]. However, this protocol has potential risks of failing to eliminate EOC completely and to reduce the risk of breast cancer due to preserving ovaries till a later stage [33]. Therefore, the development and application of non-surgical preventive approaches are needed [34]. The role of oral contraceptives, non-steroidal anti-inflammatory drugs and vitamin A analogues and VD have been investigated in reducing the risk of EOC development in the general population [35]. Nevertheless, data in *BRCA1*^mut^ carriers is limited.

We demonstrate for the first time that VD biosynthesis and/or metabolism is compromised in *BRCA1*^mut^ carriers. While *BRCA*^wt^ women had a normal VD status (44.1 ng/ml on average), *BRCA1*^mut^ carriers exhibited VD-insufficiency (29.4 ng/ml on average) according to the clinical definition (20–29.9 ng/ml serum calcifediol) [25], despite a lack of data on UV light exposure and diet supplementation. Ineffective conversion of skin-derived and dietary cholecalciferol into calcifediol in the liver may result from previously reported *BRCA1*^mut^-related liver dysfunction [36]. In addition, a prior study showed that plasma levels of VD-binding protein isotypes 1 and 2 were significantly reduced in *BRCA1*^mut^ carriers compared to *BRCA*^wt^ patients [37]. The impaired VD metabolite transportation between the skin, liver and kidney are likely to limit VD metabolism [14]. These observations have important implications for calcifediol or calcitriol, instead of cholecalciferol, as an effective VD supplementation in *BRCA1*^mut^ carriers.

VDR protein expression was reduced in OSE and FTE of healthy women with *BRCA1*^mut^ relative to *BRCA*^wt^ patients, as suggested by VDR immunostaining intensity. The difference in VDR protein expression was also observed in ovarian follicles between patient groups. Our data are consistent with *in vitro* studies showing that the number of DNA binding sites occupied by VDR are dynamically controlled by VD availability to cells [38]. Low systemic VD levels may lead to reduced VD signaling activity and diminished VDR expression in OSE, FTE and ovarian follicle granulosa cells in *BRCA1*^mut^ carriers.

The dynamic VDR expression in response to VD exposure was further examined in cultured OSE cells from healthy women with *BRCA1*^mut^. *In vitro* treatment with a relatively low dose calcitriol significantly increased VDR protein expression in OSE cells compared to that of cells cultured without calcitriol. The data are consistent with previous observation of VD-dependent VDR expression in the nonhuman primate ovary [27]. Granulosa cell VDR gene expression in cultured rhesus macaque ovarian follicles was elevated following calcitriol treatment, which improved VD actions [39]. Studies in mouse pre-osteoblastic cells [40] and human lymphoblastoid cell lines [41] have reported VDR expression and binding to its specific response DNA elements under VD-depleted conditions, which are enhanced by calcitriol treatment resulting in increased VDR occupancy and elevated downstream gene expression. Similar dynamic VDR expression could occur *in vivo* wherein the number of accessible DNA binding sites for VDR positively corelates to cellular VD stimulation. Thus, VD activity via VDR in OSE and FTE cells could be improved by VD supplementation in *BRCA1*^mut^ carriers.

The role of VD in prevention of cancers, including EOC, has remained inconclusive despite significant research effort [42]. Clinically, a tentative inverse association between circulating VD levels and ovarian cancer incidence was found by meta-analysis of existing data [19]. VD is known to regulate the expression of multiple proteins involved in the regulation of cell cycle and apoptosis [18]. Our data suggests that VD treatment may limit cell growth and/or division via maintaining cell cycle arrest. In addition, studies have previously demonstrated VD-enhanced DNA damage repair in uterine fibromas [43] and Ras oncogene-induced senescent fibroblasts [44]. Lending further support to linking VD to *BRCA1*, Deng et al. reported that *BRCA1* mediated nuclear transport of VDR and VD-induced VDR expression in rat osteosarcoma cells [45]. Campbell et al. proposed the model in which the anti-proliferative effect of VD was associated with induction of *BRCA1* gene expression via VDR in breast and prostate cancer cells [46]. Although the underlying mechanism requires further investigation, VD supplementation appears to have a potential to prevent or limit EOC development in *BRCA1*^mut^ carriers.

Supplementation of VD and calcium was found to reduce all cancer risk in postmenopausal women [47]. A randomized clinical trial showed that cancer incidence was significantly lower in patients receiving VD plus calcium supplementation as compared to the control and calcium-only supplementation groups [48]. Another study reported a 14–20% decrease in breast cancer and colorectal cancer incidence in women taking 400 IU/day calcifediol and 1000 mg/day calcium for 8 years [49]. Thus, investigating the effect of VD supplementation on EOC incidence in *BRCA1*^mut^ carriers will further the knowledge on precise role of VD in EOC prevention.

Studies on VD-associated cancer prevention have generated conflicting results due to the limitations of combining different types of cancers which are heterogeneous and arise from the dysregulation of multiple cellular processes. Our study focuses on VD action in *BRCA1*^mut^ carriers using biological materials from healthy women with distinct *BRCA1*^mut^. Studies of VD action are limited to *in vitro* experiments using cell culture, which show promising results. Experiments in animal models with a *BRCA1*^mut^ background are needed to further explore VD effects on cancer development *in vivo*.

Collectively, our results suggest a potential chemo-preventive role of VD in EOC development in women with *BRCA1*^mut^. Future studies will investigate the relationship between impaired VD action and EOC incidence in *BRCA1*^mut^ carriers, as well as the mechanism and role of VD in EOC prevention in women with *BRCA1*^mut^. The availability of a chemo preventive strategy for *BRCA1*^mut^ carriers, such as VD supplementation, will be a significant improvement on current risk reducing strategies. As mutations in *BRCA1* gene also result in an increased risk of breast cancer [9], our findings may provide important implications for breast cancer prevention in *BRCA1*^mut^ carriers.

## MATERIALS AND METHODS

### Patient sample collection

Ovarian and fallopian tube tissue samples and corresponding blood samples, as well as clinical information, were obtained from the Oregon Ovarian Cancer Registry and Tissue Repository (OOCRTR) and the Oregon Clinical and Translational Research Institute (OCTRI) at the Oregon Health & Science University (OHSU). All samples were collected with informed consent, and the experimental protocol was approved by the Institutional Review Board (IRB; #921 and #3485).

### Systemic VD levels in patients

Systemic VD levels of patients were determined using electrochemiluminescence immunoassay. Blood samples were collected from heathy pre-menopausal (≤ 45 years old) women with *BRCA1*^mut^ (*n* = 11) (Table 1) and women without *BRCA* mutations (*BRCA*^wt^; *n* = 13). Serum calcifediol (major circulating form of VD) concentrations were measured using a Cobas Elecsys kit (catalog number: 06506780160; Roche Diagnostics, Indianapolis, IN) by the Endocrine Technologies Core at the Oregon National Primate Research Center, OHSU, as previously described [39].

### VDR expression in OSE and FTE

VDR expression in OSE and FTE were evaluated using immunohistochemistry, as previously reported [27]. Ovarian and fallopian tube tissues were collected from *BRCA*^wt^ patients (*n* = 5 for the ovary; *n* = 3 for the fallopian tube) and *BRCA1*^mut^ carriers (*n* = 4) (Table 1), and fixed for paraffin embedding. Deparaffinized 5 μm sections were rehydrated in ethanol, followed by incubation at 4°C overnight with mouse anti-human VDR antibody (1:200; sc-13133; Santa Cruz Biotechnology, Inc., Santa Cruz, CA). Phosphate-buffered saline was used instead of the first antibody as negative control. Sections were then incubated with the secondary antibody and processed using the ImmPress HRP Reagent Kit (MP-7402, Vector Laboratories, Burlingame, CA). The antigen-antibody complex was visualized by incubation with 3,3′-diaminobenzidine. Ovarian tissue sections were counterstained using hematoxylin to demonstrate the nuclear versus cytoplasmic VDR staining. Images were captured via an Olympus BX40 inverted microscope and an Olympus DP72 digital camera (Olympus Imaging America Inc., Center Valley, PA, USA).

### OSE cell culture

OSE cells from healthy women with *BRCA1*^mut^ (*n* = 3) (Table 1) who underwent risk-reducing bilateral salpingo-oophorectomy were kindly provided by Dr. Elizabeth Swisher, University of Washington, Seattle, WA. Cells were cultured in defined medium: 50/50 (v/v) DMEM/RPMI 1640 supplemented with 20% (v/v) fetal bovine serum, epidermal growth factor (0.01 μg/ml), gentamycin (50 μg/ml), ciprofloxacin (10 μg/ml), insulin (10 μg/ml) and penicillin/streptomycin (100 μg/ml) (Sigma-Aldrich, St. Louis, MO) in 5% CO_2_ at 37°C. Cells grew to approximately 80% confluence prior to harvesting.

### VDR expression in cultured OSE cells

OSE cells (*n* = 3 patients) were plated at 100,000 cells/well in a 6-well plate. Twenty-four hours post plating, cells were treated with 0 (control) or 5 nM calcitriol (biologically active form of VD; Sigma-Aldrich) supplementation in the culture medium for 72 hrs. Cells were harvested for Western blot, as previously described [50]. Briefly, total protein was extracted using a RIPA buffer containing 150 mM NaCl, 1% (v/v) NP-40, 1% (w/v) sodium deoxycholate, 0.1% (w/v) SDS, and 25 mM Tris-HCl (pH 7.6), electrophoresed on 4–12% Bis-Tris gel, and transferred to a nitrocellulose membrane. The membrane was blocked with 5% (w/v) nonfat milk in 10 mM Tris-buffered saline (pH 8.0), and then incubated with mouse anti-human VDR (1:250; sc-13133; Santa Cruz Biotechnology, Inc.) or α-Tubulin (loading control; 1:24,000; T6074; Sigma-Aldrich) antibody at 4°C overnight. The membrane was subsequently incubated with horseradish peroxidase-conjugated secondary antibody (Abcam, Burlingame, CA, USA). Images developed on the film were scanned using the Konica Minolta Bizhub C368 (Konica Minolta, Wayne, NJ, USA). Densitometry analysis was performed using ImageJ software (National Institutes of Health, Bethesda, MD, USA).

### Viability of cultured OSE cells

OSE cells (*n* = 3 patients) were plated at 5,000 cells/well in a 96-well plate. Twenty-four hours post plating, cells were treated with 0 (control) or 5 nM calcitriol supplementation in the culture medium for 72 hrs. Cell viability was assessed using the LIVE/DEAD Viability/Cytotoxicity Kit (Catalogue # 10237012, Invitrogen, Carlsbad, CA, USA) according to the manufacturer’s directions. Briefly, cells were incubated with 1 μM calcein AM and 2 μM EthD-1 at room temperature for 30 min. Images were captured using an EVOS FL imaging system (Themo Fisher Scientific, Waltham, MA, USA). All experiments were carried out in triplicate.

### Proliferation of cultured OSE cells

OSE cells (*n* = 3 patients) were plated at 100,000 cells/well in a 6-well plate. Twenty-four hours post plating, cells were treated with 0, 0.1, 1 or 10 nM calcitriol supplementation in the culture medium for 72 hrs. Cells were then harvested and counted using a hemocytometer under a Leica DM IL microscope (Leica, Wetzler, Germany). All experiments were carried out in triplicate.

### Statistical analysis

Data involving two and multiple experimental groups were analyzed by a Student’s *t*-test and one-way analysis of variance, respectively, using SigmaPlot 11 software (SPSS, Chicago, IL).
